# Supplementary material for: Differential expression of TKS4 isoforms and their role in cellular processes in breast cancer
Source: J Cell Commun Signal. 2026 Aug 18;20(3):e70100. doi: 10.1002/ccs3.70100 (PMC13486968; doi:10.1002/ccs3.70100)
Supplement: Supplementary file 1 [file CCS3-20-e70100-s001.docx]

**Supplementary Materials**

Differential Expression of TKS4 Isoforms and Their Role in Cellular Processes in Breast Cancer

Serhii Kropyvko, Nazar Shevchuk, Olga Gubar, Kyrylo Lavrynenko, Yaroslav Nemesh, Denys Kozakov, Valentin Polischuk, Valentyna Kryklyva, Liubov Syvak, Nataliia Verovkina, Tetyana Gryaznova

**Table S1. Commercial antibodies to TKS4**, related to Introduction

| Name | Firm | Catalog number | Host species | Monoclonal/polyclonal | Immunogen region/epitope |
| --- | --- | --- | --- | --- | --- |
| Anti-Sh3Pxd2B Antibody Produced In Rabbit * | Sigma Aldrich | Cat#HPA058141 | Rabbit | Polyclonal | aa 431-532 |
| SH3PXD2B Polyclonal Antibody | Invitrogen | #PA5-57673 | Rabbit | Polyclonal | aa 556-642 (#[RP-96944](https://www.thermofisher.com/order/catalog/product/RP-96944)) |
| SH3PXD2B Antibody | [Novus Biologicals](https://www.novusbio.com/guarantee) | NBP3-25137 | Rabbit | Polyclonal | aa 431-532 |
| Anti-SH3PXD2B antibody | Abcam | ab122342 | Rabbit | Polyclonal | aa 550-650 |
| Anti-SH3PXD2B | [Atlas Antibodies](https://www.antibodypedia.com/supplier_details.php?supplier_id=1) | HPA036471 | Rabbit | Polyclonal | aa 556-642 |
| SH3PXD2B polyclonal antibody | [Abnova Corporation](https://www.antibodypedia.com/supplier_details.php?supplier_id=238) | PAB23008 | Rabbit | Polyclonal | aa 556-642 |
| SH3PXD2B antibody (AA 300-350) | Antibodies online | ABIN7451528 | Rabbit | Polyclonal | aa 300-350 |
| SH3PXD2B antibody (AA 505-539) | Antibodies online | ABIN6244203 | Rabbit | Polyclonal | aa 505-539 |
| SH3PXD2B antibody (AA 596-628) | Antibodies online | ABIN6244202 | Rabbit | Polyclonal | aa 596-628 |
| Polyclonal Rabbit anti‑Human SH3PXD2B Antibody (aa300‑350) | LS Bio | LS‑C290107 | Rabbit | Polyclonal | aa 300-350 |
| anti-SH3PXD2B antibody | MyBioSource | [MBS6499363](https://www.mybiosource.com/polyclonal-sh3pxd2b-human-mouse-antibody/sh3-and-px-domain-containing-protein-2b/6499363) | Rabbit | Polyclonal | aa 257-477 |

* - antibodies used in this work

**Table S2. Ratios of *TKS4* gene transcript expression in human cell lines**, related to Figures 2.

| **Human cell lines** | ***TKS4L/TKS4b*** | ***TKS4L/TKS4d*** | ***TKS4b/TKS4d*** |
| --- | --- | --- | --- |
| 293 | 0.716 | 3.201 | 4.47 |
| KRC/Y | 0.42 | 0.354 | 0.843 |
| HeLa | ― | ― | ― |
| U-251 MG | 2.031 | 8.835 | 4.348 |
| U-87 MG | 0.923 | 2.05 | 2.221 |
| T98G | 0.939 | 4.357 | 4.637 |
| PNT2 | 1.54 | 4.543 | 2.949 |
| LNCaP | 1.988 | 4.196 | 2.11 |
| DU145 | 0.671 | 0.671 | 1 |
| PC-3 | 1.074 | 6.724 | 5.015 |
| SH-SY5Y | 2.544 | 37.867 | 14.883 |
| ARPE-19 | 0.615 | 2.855 | 4.299 |
| SKOV-3 | 0.887 | 2.327 | 2.624 |
| Hep G2 | 1.802 | 3.31 | 1.836 |
| HT-29 | 1.031 | 1.777 | 2.27 |
| A549 | 0.665 | 1.534 | 2.304 |
| JKT-1 | 1.9 | 3.08 | 1.613 |
| T-47D | 6.078 | 13.811 | 2.272 |
| MCF-7 | 2.633 | 7.56 | 2.87 |
| MDA-MB-231 | 1.063 | 1.338 | 1.257 |
| HaCat | 2.639 | 4.205 | 1.593 |
| A431 | ― | 4.042 | ― |
| WM793 | 2.05 | 5.275 | 2.572 |
| T24 | 0.455 | 0.849 | 1.866 |
| 5637 | 1.817 | 5.477 | 3.013 |

**Figure S1. *TKS4* expression in normal and tumor samples in various cancer types based on the GENT2 database – full version,** related to Figures 3A. The BC is highlighted with a box.

**Table S3.** **Statistical analysis of *TKS4* gene expression in different tumor types based on the GENT2 database**, related to Figures 3A, S1.

| **Tissue** | **p-value** | **Log2FC** |
| --- | --- | --- |
| All | <0.001 | 0.120 |
| Adipose | 0.003 | -0.977 |
| Adrenal Gland | 0.004 | -0.546 |
| Bladder | 0.229 | 0.187 |
| Blood | <0.001 | 0.413 |
| Bone | 0.032 | 0.982 |
| Brain | <0.001 | 0.715 |
| Breast * | 0.196 | -0.062 |
| Cervix | 0.459 | -0.216 |
| Colon | <0.001 | 1.094 |
| Endometrium | <0.001 | -0.801 |
| Esophagus | 0.001 | -0.652 |
| Eye | NA | -0.649 |
| Gallbladder | 0.458 | 0.727 |
| Head and Neck | 0.193 | 0.219 |
| Kidney | <0.001 | 0.774 |
| Liver | <0.001 | 0.892 |
| Lung | <0.001 | 0.159 |
| Lymph Node | NA | 1.205 |
| Muscle | 0.309 | 1.423 |
| Oral | 0.008 | -0.934 |
| Ovary | <0.001 | 0.998 |
| Pancreas | <0.001 | 0.475 |
| Pharynx | 0.025 | -0.890 |
| Prostate | 0.012 | -0.405 |
| Skin | <0.001 | -0.343 |
| Small Intestine | 0.890 | -0.057 |
| Spleen | 0.735 | 0.246 |
| Stomach | <0.001 | 0.379 |
| Teeth | <0.001 | 1.139 |
| Testis | 0.004 | -0.950 |
| Thyroid | <0.001 | 0.727 |
| Tongue | 0.022 | 0.564 |
| Uterus | 0.759 | -0.051 |
| Vagina | 0.215 | -0.856 |
| Vulva | 0.492 | 0.141 |

Listed in descending order of Log2FC. The type of cancer that was further investigated, highlighted with a star.

**Figure S2.** **Expression of the *TKS4L*, *TKS4b*, and *TKS4d* transcripts in normal and breast tumor tissues based on analysis of the TCGA dataset using the UCSC Xena browser**, related to RESULTS «**Expression profile of *TKS4* transcripts in breast tumors**».

**Table S4.** **Expression of the *TKS4* gene in BC tumors**, related to Figures 3D.

| **Types of human BC** | ***TKS4*** | **CI** | **p-value** | ***TKS4L*** | **CI** | **p-value** | ***TKS4b*** | **CI** | **p-value** |
| --- | --- | --- | --- | --- | --- | --- | --- | --- | --- |
| LumA | 1.338 | 1.16-1.20 | 0.092 | 3.731 | 3.20-4.35 | 0 | 1.017 | 0.85-1.21 | 0.949 |
| LumB HER2– | 0.899 | 0.78-1.03 | 0.577 | 3.098 | 2.58-3.72 | 2.3*10^-5^ | 0.55 | 0.44-068 | 0.036 |
| Triple Negative | 0.856 | 0.75-0.97 | 0.400 | 2.958 | 2.40-3.64 | 1.6*10^-4^ | 0.613 | 0.51-0.73 | 0.062 |
| LumB HER2+ | 1.064 | 0.94-1.20 | 0.744 | 2.608 | 2.08-3.27 | 5.4*10^-4^ | 0.63 | 0.52-0.75 | 0.077 |
| HER2-Enriched | 1.580 | 1.38-1.80 | 0.018 | 4.075 | 3.23-5.14 | 1.6*10^-5^ | 0.603 | 0.51-0.71 | 0.041 |

Fold change in the expression of the total pool of *TKS4* transcripts, as well as individual *TKS4L* and *TKS4b* transcripts, in different human BC tumor types compared with adjacent tissues. The p-value for the means was calculated via Fisher's criterion. CI – confidence interval.

**Table S5. Ratios of *TKS4* transcripts expression in human BC tumor types and adjacent tissues**, related to Figures 3E.

| **Types of human BC** | ***TKS4L/TKS4b*** | ***TKS4b/TKS4L*** |
| --- | --- | --- |
| Adjacent | 0.21 | 4.76 |
| LumA | 0.67 | 1.48 |
| LumB HER2– | 1.2 | 0.84 |
| Triple Negative | 0.89 | 1.13 |
| LumB HER2+ | 0.78 | 1.27 |
| HER2-Enriched | 1.28 | 0.78 |

**Figure S3.** **The TKS4 isoforms are not recognized by the SH2 domains without overexpression Src(Y527F)**, related to Figures 5B. Analysis of TKS4L and TKS4b isoform interactions with the GST-SН2 domains of PI3K, FYN, FGR, ITK, ABL, PLCg1, NCK1, NCK2, CRKI, GRB2 proteins and GST alone (control), which were immobilized on glutathione beads and incubated with lysates from growing T-REx-293 cells with transiently expressed omni-TKS4b. Bound proteins were analyzed by Western blotting using antibodies against TKS4L, anti-omni and anti-GST antibodies. WB, Western blot.

**Figure S4.** **Proliferation rates of the cell lines MCF-7wt, MCF-7ΔTKS4, MCF-7+TKS4L, and MCF-7+TKS4b (M±σ, n=6)**, related to Table 1**.**

**Table S6.** **Genes expression in TKS4-cell lines**, related to Figures 6C-D.

| **mRNA name** | **MCF-7ΔTKS4** | **MCF-7+TKS4L** | **MCF-7+TKS4b** |
| --- | --- | --- | --- |
| *TKS4L* | 0.29*** | 137.937*** | 0.578*** |
| *TKS4b* | 0.249*** | 0.3*** | 8693.201*** |
| *TKS4d* | 0.219*** | 1.03 | 0.722 |
| *TKS5* | 0.000018*** | 0.649** | 0.603** |
| *TKS5L* | 0.048*** | 0.257*** | 0.407*** |
| *WIP* | 0.739** | 0.241*** | 0.473*** |
| *WASL* | 0.197*** | 0.033*** | 0.163*** |
| *CR16* | 0.075*** | 0.272*** | 0.678** |
| *TTP* | 2.711** | 0.309*** | 0.4** |

Fold change in the expression of *TKS4L*, *TKS4b*, *TKS4d*, *TKS5*, *TKS5L*, *WIP*, *WASL*, *CR16* and *TTP* transcripts in MCF-7ΔTKS4, MCF-7+TKS4L, and MCF-7+TKS4b cells compared with that in MCF-7wt cells. The p-value for the means was calculated via Fisher's criterion. * – *p* < 0.05, ** – *p* < 0.01, *** – *p* < 0.001

**Figure S5.** **Hypothetical interaction of the TKS4L and TKS4b isoforms after phosphorylation**, related to Discussion. Hypothetical schemes for the formation of homo- and heterodimers of the TKS4L and TKS4b isoforms after phosphorylation, assuming interaction between the SH3_1 and SH3_2 domains and the TAM motif of another molecule.

**Figure S6.** **Western blot analysis of the MCF-7 cell lines with knockout and overexpression of TKS4.** Related to METHODS «Generation of MCF-7 stable cell lines with a knockout and overexpression of *TKS4* gene». **(A)** WB analysis of *TKS4* gene knockout in MCF-7ΔTKS4 cell clones. *TKS4* gene was edited with CRISPR/Cas9 technology. SH3PXD2B antibodies against the C-terminal region of TKS4 were used for the analysis. β-Actin was used as a loading control; **(B)** WB analysis of TKS4L-myc overexpression in MCF-7+TKS4L cell line; **(C)** WB analysis of TKS4b-omni overexpression in MCF-7+TKS4b cell line. TKS4L-myc and TKS4b-omni overexpression was performed with pcDNA4/TO methodology.

**Table S7. Cell lines**. Related to METHODS «Cell culture and transfection».

| **Official cell line name** | **Species** | **Sex** | **Tissue of origin** | **Source** | **Research Resource Identifier (RRID)** |
| --- | --- | --- | --- | --- | --- |
| U-87 MG | Human | Male | Brain | ATCC | HTB-14 |
| U-251 MG | Human | Male | Brain | ATCC | RRID: CVCL_0021 |
| T98G | Human | Male | Brain | ATCC | CRL-1690 |
| HEK-293 | Human |  | Kidney; Embryo | ATCC | CRL-1573; RRID: CVCL_0045 |
| KRC/Y | Human | Female | Kidney | Cellosaurus | RRID: CVCL_A448 |
| HeLa | Human | Female | Uterus; Cervix | ATCC | CCL-2; RRID: CVCL_0030 |
| ARPE-19 | Human | Male | Eye; Retina; Retinal pigmented epithelium | ATCC | CRL-2302; RRID: CVCL_0145 |
| T-47D | Human | Female | Breast; Mammary gland | ATCC | HTB-133, RRID: CVCL_0553 |
| MCF-7 | Human | Female | Breast; Mammary gland | ATCC | HTB-22, RRID: CVCL_0031 |
| MDA-MB-231 | Human | Female | Breast; Mammary gland | ATCC | CRM-HTB-26 |
| SH-SY5Y | Human | Female | Bone; Marrow | ATCC | CRL-2266 |
| SKOV-3 | Human | Female | Ovary; Ascites | ATCC | HTB-77 |
| HT1080 | Human | Male | Connective tissue | ATCC | CCL-12 |
| HaCaT | Human | Male | Skin | Cytiom | 300493, RRID: CVCL_0038 |
| A431 | Human | Female | Skin; Epidermis | ATCC | CRL-1555 |
| WM793 | Human | Male | Skin | Cellosaurus | RRID: CVCL_8787 |
| Hep G2 | Human | Male | Liver | ATCC | HB-8065, RRID:CVCL_0027 |
| HT-29 | Human | Female | Colon | ATCC | HTB-38, RRID: CVCL_0320 |
| A549 | Human | Male | Lung | ATCC | CRM-CCL-185, RRID: CVCL_0023 |
| JKT-1 | Human | Male | Testis | Cellosaurus | RRID: CVCL_T011 |
| PNT2 | Human | Male | Prostate | ECACC | 95012613; RRID: CVCL_2164 |
| DU145 | Human | Male | Prostate | ATCC | HTB-81, CVCL_0105; RRID: CVCL_0105 |
| LNCaP | Human | Male | Prostate | ATCC | CRL-1740, RRID: CVCL_0395 |
| PC-3 | Human | Male | Prostate | ATCC | PC-3, RRID: CVCL_0035 |
| 5637 | Human | Male | Urinary bladder | ATCC | HTB-9, RRID: CVCL_0126 |
| T24 | Human | Female | Urinary bladder | ATCC | HTB-4, RRID: CVCL_0554 |
| Flp-In™ T-REx™-293 | Human |  | Kidney; Embryo | Invitrogen | R780-07 |
| T-REx-293-omni-TKS4b | Human |  | Kidney; Embryo | This study | N/A |
| T-REx-293-myc-Src(Y527F) | Human |  | Kidney; Embryo | This study | N/A |
| MCF-7ΔTKS4 | Human | Female | Breast; Mammary gland | This study | N/A |
| MCF-7+TKS4L | Human | Female | Breast; Mammary gland | This study | N/A |
| MCF-7+TKS4b | Human | Female | Breast; Mammary gland | This study | N/A |

**Table S8 Antibodies**. Related to METHODS «Antibodies and immunofluorescence reagents».

| **Antibodies** | **Source** | **Research Resource Identifier (RRID)** |
| --- | --- | --- |
| Anti-omni (D-8) mouse | Santa Cruz | Cat# sc-7270; RRID: AB_675763 |
| Anti-omni (M-21) rabbit | Santa Cruz | Cat# sc-499; RRID: AB_675765 |
| Anti-Vimentin (V9) mouse | Santa Cruz | Cat# sc-6260; RRID: AB_628437 |
| Anti-SMA (Smooth Muscle Actin) (CGA7) mouse | Santa Cruz | Cat# sc-53015; RRID: AB_628683 |
| Anti-myc (9E10) mouse | Santa Cruz | Cat# sc-40; RRID: AB_627268 |
| Anti-phosphotyrosine (PY99) mouse | Santa Cruz | Cat# sc-7020 |
| Anti-flag M2 mouse | Sigma Aldrich | Cat# F1804; RRID: AB_262044 |
| Anti-β-actin mouse | Sigma Aldrich | Cat# A1978; RRID: AB_476692 |
| Anti-α-tubulin mouse | Sigma Aldrich | Clone B-5-1-2; Cat# T6074 |
| Anti-SH3PXD2B rabbit | Sigma Aldrich | Cat# HPA058141 |
| Anti-GST goat | Sigma Aldrich | Cat# GE27-4577-01 |
| Anti-N-Cadherin rabbit | Abcam | Cat# ab18203; RRID: AB_444317 |
| Anti-E-Cadherin rabbit | Abcam | Cat# ab40772; RRID: AB_731493 |
| Anti-CTTN (p80/85) mouse | Sigma Aldrich | Clone 4F11; Cat# 05-180-I |
| Goat anti-rabbit IgG, HRP Conjugate | Promega | Cat# W4011; RRID: AB_430833 |
| Goat anti-mouse IgG, HRP Conjugate | Promega | Cat# W4021; RRID: AB_430834 |
| Donkey anti-goat IgG, HRP Conjugate | Promega | Cat# V8051; RRID: AB_430838 |
| Goat anti-mouse Alexa Fluor 633 | Thermo Fisher | Cat# A-21050; RRID: AB_2535718 |
| Donkey anti-rabbit Alexa Fluor 555 | Abcam | Cat# ab150074; RRID: AB_2636997 |
| Goat anti-mouse Alexa Fluor 488 | Invitrogen | Cat# A-11001; RRID: AB_2534069 |
| Alexa Fluor 555-phalloidin | Invitrogen | Cat# A34055 |

**Table S9. Primers and Taq-Man probes used for qPCR**. Related to METHODS «cDNA synthesis and qPCR with fluorescence labeled probes».

| **Gene name,**  **GenBank accession number of mRNA** | ***Sequences with positions on the mRNA** | ****Primer** **efficacy,**  **%** |
| --- | --- | --- |
| human *TBP*  NM_003194.5 | for. 800-820 5’gtgcccgaaacgccgaatata3’  Taq-Man probe 821-842 5’(BHQ1)atcccaagcggtttgctgcggt(FAM)3’  rev. 874-854 5’ccgtggttcgtggctctctta3’; | 91.5 |
| human *TKS4*  NM_001017995 | for. 226-250 5’cgggtgcccaacaagcattatgtct3’  Taq-Man probe 279-304 5’(ROХ)ctccaccgaggccatttaccggcgct(BHQ2)3’  rev. 352-374 5’gggtccttctgtcctccttccat3’ | 72.8 |
| human *TKS4L*  NM_001017995 | for. 1286-1305 5’agtattacaccatcgccgaa3’  Taq-Man probe 1320-1339 5’(HEX)cccagacggcatcagcttcc(BHQ1)3’  rev. 1365-1386 5’ccagccactcaagtttttctcg3’ | 104.2 |
| human *TKS4b*  NM_001308175.1 | for. 1286-1305 5’agtattacaccatcgccgaa3’  Taq-Man probe 1320-1339 5’(HEX)cccagacggcatcagcttcc(BHQ1)3’  rev. 1369-1388 5’gggctctccaagtgaacatt3’ | 94.3 |
| human *TKS4d* | for. 5’agtattacaccatcgccgaa3’  Taq-Man probe 5’(HEX)cccagacggcatcagcttcc(BHQ1)3’  rev. 5’atctccagcgtcatcctat3’ | 91.7 |
| human *TKS5L*  NM_014631 | for. 239-262 5’tgactccacctcccagactatcta3’  Taq-Man probe 54-379 5’(FAM)atccccttcctcccaggcaagatcct(BHQ1)3’  rev. 407-431 5’gatgggcttcagtctcttcacagct3’ | 93.7 |
| human *TKS5*  NM_014631 | for. 955-974 5’tggaaggctggtggtatatc3’  Taq-Man probe 982-1001 5’(FAM)cgcccagccctctttgccca(BHQ1)3’  rev. 1005-1023 5’ccttcttcaggtaggatgc3’ | 86.7 |
| human *CR16*  NM_001080529 | for. 1494-1513 5’aaaccatgccagaagattta3’  Taq-Man probe 1524-1543 5’(HEX)atccccagaagccgtacacc(BHQ1)3’  rev. 1587-1606 5’gaatttctgcctttgatgtc3’ | 91.4 |
| human *WIP*  NM_001077269 | for. 1597-1616 5’aactggcaagaaacgaaagc3’  Taq-Man probe 1646-1669 5’(ROХ)tgctccaccactccctcccatccc(BHQ2)3’  rev. 1673-1692 5’gaagagcaggcaaagatcac3’ | 89 |
| human *WASL*  NM_003941.3 | for. 1147-1166 5’tgaactgcggaggcaagcac3’  Taq-Man probe 1198-1220 5’(FAM)acctcctcctcctccccctccac(BHQ1)3’  rev. 1247-1265 5’gagcgcctcttcccctagc3’ | 98 |
| human *TTP*  NM_003407.5 | for. 58-79 5’catggatctgactgccatctac3’  Taq-Man probe 98-117 5’(FAM)agccctgacgtgcccgtgcc(BHQ1)3’  rev. 177-195 5’ctggagtcggaggggctca3’ | 102.1 |

* – Primer and Taq-Man probe (Metabion) design was performed with OLIGO Version 7.60.

** – PCR efficacy was determined via LinRegPCR Version 2021.2 software.
